# A Case-Only Study of Vulnerability to Heat Wave–Related Mortality in New York City (2000–2011)

**DOI:** 10.1289/ehp.1408178

**Published:** 2015-03-17

**Authors:** Jaime Madrigano, Kazuhiko Ito, Sarah Johnson, Patrick L. Kinney, Thomas Matte

**Affiliations:** 1 Department of Environmental and Occupational Health, School of Public Health, Rutgers, The State University of New Jersey, Piscataway, New Jersey, USA; 2 Bureau of Environmental Surveillance and Policy, New York City Department of Health and Mental Hygiene, New York, New York, USA; 3 Department of Environmental Health Sciences, Mailman School of Public Health, Columbia University, New York, New York, USA

## Abstract

**Background:**

As a result of climate change, the frequency of extreme temperature events is expected to increase, and such events are associated with increased morbidity and mortality. Vulnerability patterns, and corresponding adaptation strategies, are most usefully conceptualized at a local level.

**Methods:**

We used a case-only analysis to examine subject and neighborhood characteristics that modified the association between heat waves and mortality. All deaths of New York City residents from 2000 through 2011 were included in this analysis. Meteorological data were obtained from the National Climatic Data Center. Modifying characteristics were obtained from the death record and geographic data sets.

**Results:**

A total of 234,042 adult deaths occurred during the warm season of our study period. Compared with other warm-season days, deaths during heat waves were more likely to occur in black (non-Hispanic) individuals than other race/ethnicities [odds ratio (OR) = 1.08; 95% CI: 1.03, 1.12], more likely to occur at home than in institutions and hospital settings (OR = 1.11; 95% CI: 1.06, 1.16), and more likely among those living in census tracts that received greater public assistance (OR = 1.05; 95% CI: 1.01, 1.09). Finally, deaths during heat waves were more likely among residents in areas of the city with higher relative daytime summer surface temperature and less likely among residents living in areas with more green space.

**Conclusion:**

Mortality during heat waves varies widely within a city. Understanding which individuals and neighborhoods are most vulnerable can help guide local preparedness efforts.

**Citation:**

Madrigano J, Ito K, Johnson S, Kinney PL, Matte T. 2015. A case-only study of vulnerability to heat wave–related mortality in New York City (2000–2011). Environ Health Perspect 123:672–678; http://dx.doi.org/10.1289/ehp.1408178

## Introduction

As a result of climate change, the frequency of extreme heat days is expected to increase, and such events are associated with increased morbidity and mortality (Anderson and Bell 2011; Armstrong 2006; Braga et al. 2001; Zanobetti and Schwartz 2008). Although much of the research on climate change has been done on a large spatial scale, it is increasingly recognized that vulnerability patterns and corresponding adaptation strategies are most usefully conceptualized at a local level. To allocate resources efficiently, local governments need to understand what population and neighborhood characteristics will increase vulnerability.

Recognizing that neighborhood contextual factors increase risk of heat-related morbidity and mortality, mapping studies have demonstrated spatial variability in heat vulnerability. These studies typically map physical determinants of heat risk (e.g., temperature exposure and duration) (Kershaw and Millward 2012), a set of vulnerability characteristics that are expected to contribute to heat-related morbidity or mortality based on prior epidemiology studies (Reid et al. 2009), or both (Buscail et al. 2012). However, they do not link these vulnerability characteristics with observed health outcomes.

In contrast, the epidemiologic literature uses observed individual-level health outcome data to determine the relative increase in risk due to a set of vulnerability characteristics. Such single- (Schwartz 2005) and multi-city studies have been conducted (Zanobetti et al. 2013). However, few studies within (Hondula et al. 2012; Uejio et al. 2011) and outside the United States (Kosatsky et al. 2012; Xu et al. 2013) have used observed heat-related health outcomes to inform vulnerability mapping, and those within the United States have been based on extreme heat case definitions (which rely on body temperature and environmental conditions) or ZIP code area–level characteristics.

In the United States, few metropolitan areas are as diverse and densely populated as New York City (NYC). This setting provides a unique opportunity to examine individual and small-area (census tract level) characteristics that increase vulnerability to extreme heat. Such an analysis can be used to create a vulnerability map that will help guide future public health prevention and preparedness efforts. Excess mortality due to heat waves has already been demonstrated in NYC (Metzger et al. 2010), and an ecological analysis demonstrated that area-level rates of heat-associated mortality of seniors in NYC were correlated with prevalence of poor housing conditions, poverty, hypertension, impervious land cover, and high land surface temperatures (Klein Rosenthal et al. 2014). We therefore conducted a case-only analysis to examine whether heat-related mortality risk varied according to individual and neighborhood characteristics and used these results to inform the creation of a composite vulnerability index for NYC.

## Methods

The case-only design was originally proposed to study gene–environment interactions (Hamajima et al. 1999; Khoury and Flanders 1996). In 2003, Armstrong proposed that such an approach could be used to investigate how characteristics that do not vary (or vary slowly) over time modify the effect of a time-varying environmental exposure on an outcome (Armstrong 2003). Here, we apply this method to examine vulnerability to heat-related mortality in NYC. Modifiers of interest were obtained from the death certificate record and various geographic data sets described below.

*Meteorological data*. Meteorological data were obtained from the National Climatic Data Center (National Oceanic and Atmospheric Administration, National Centers for Environmental Information 2012) for the station at LaGuardia Airport in NYC because this station had the most complete data of the three NYC stations. As in previous studies of heat waves and mortality in NYC (Metzger et al. 2010), heat index was calculated using ambient temperature and relative humidity (Steadman 1979). For this analysis, we defined heat wave days as those days when the maximum temperature or maximum heat index exceeded 95°F for at least 2 consecutive days. This definition is consistent with available variations of heat indices (Perkins and Alexander 2013) and consistent with findings in NYC that mortality increased nonlinearly with lags (1–2 days) when the maximum heat index reached 90–100°F, and this exposure metric worked as well or better than alternative metrics (Metzger et al. 2010).

*Mortality data*. We obtained data on every death occurring in NYC of an adult (> 19 years of age) resident for the period 2000 through 2011 from the NYC Department of Health and Mental Hygiene (NYC DOHMH) Office of Vital Statistics. Data included underlying cause of death, age, sex, race/ethnicity, country of birth, place of death, and census tract of residence. Underlying causes of death [and *International Classification of Diseases, 10th Revision* (ICD-10) codes] of particular interest included cardiovascular disease (I00–I99), myocardial infarction (I21), congestive heart failure (I50), and chronic obstructive pulmonary disease (J40–J47). The NYC DOHMH Institutional Review Board (IRB) approved this study, and the Columbia University IRB deemed it was not human subjects research. We restricted the analysis to nonexternal-cause mortality (ICD-10, A00-R99; ICD-9, 001–799) during the warm season (May–September).

*Neighborhood data*. Neighborhood data was assigned according to residential census tract. Deaths occurring between 2000 and 2009 had corresponding residential addresses based on Census 2000 and deaths from 2010 through 2011 based on Census 2010. Census tracts have an average population of about 4,000 and are designed to have relatively homogeneous socioeconomic characteristics. In NYC, the average land area of census tracts is about 90 acres (NYC Census FactFinder 2013). Census tract area–based socioeconomic measures have been found to be good proxies for individual estimates when examining health inequalities (Subramanian et al. 2006). We obtained data on neighborhood characteristics including the proportion of families receiving public assistance and the proportion of non-English–speaking residents from the Census 2000 (U.S. Census Bureau 2001) for all tracts (*n* = 2,216). Building density and land-use data were obtained from the NYC Department of City Planning (DCP) Primary Land Use Tax Lot Output for calendar year 2011 (DCP 2011) and aggregated to Census Tract 2000. The proportion of area covered by trees, grass, and shrubs for a given census tract was calculated from a LIDAR-based Land Cover classified surface (MacFaden et al. 2012). Census tract estimates of all-vehicle and truck-only traffic densities were obtained from the New York Metropolitan Transportation Council (NYMTC)–modeled traffic data for calendar year 2005 (NYMTC 2005). Finally, relative spatial variability in temperature was estimated through two different approaches to capture daytime surface temperature and overnight air temperature. For daytime temperature, census tract minimum, maximum, and mean surface temperature were calculated from LandSat thermal band images (U.S. Geological Survey 2009) for 18 August 2009 at 1522 hours (a day with a maximum temperature of 90^o^F and 3% cloud cover). To examine variability in overnight cooling, we calculated census tract minimum, maximum, and mean temperature using ambient summer minimum temperature (0300–0500 hours) created from predictions from land-use regression modeling of average street-level temperature data collected at 100 locations across NYC during 2009 and 2010 as part of the New York City Community Air Survey (Clougherty et al. 2013; Matte et al. 2013). For deaths corresponding to Census 2010 tracts that differed from Census 2000 tracts, we assigned neighborhood characteristics from Census 2000 boundaries equally if the tract was split (*n* = 54) and by averaging if tracts were combined (*n* = 94).

*Statistical analysis*. If a characteristic increases the risk of dying on hotter days, the proportion of deaths with that characteristic will be higher on hotter days than on other days. In this analysis, we used logistic regression to examine whether binary modifiers of interest were associated with an increased odds of death during heat wave days compared with non–heat wave days during the warm season. We first considered deaths exposed only if they occurred during heat wave days, and subsequently considered deaths that occurred during the heat wave as well as 2 days immediately following the heat wave. Individuals were defined as either having the modifier (characteristic or condition) or not, based on the death certificate data. Neighborhood modifiers were dichotomized at their median value, such that census tracts were classified as either high or low for a given neighborhood characteristic. All statistical analysis was conducted using PROC LOGISTIC in SAS version 9.2 (SAS Institute Inc., Cary, NC). Although the main effect of season drops out in a case-only analysis, confounding can occur if there is an interaction between season and the modifier of interest. Therefore, we performed a sensitivity analysis with the addition of a sine and cosine term to the models to model seasonality. We performed additional sensitivity analyses dichotomizing modifiers at the 75th percentile and using multinomial logistic regression to look at categories of certain modifiers (e.g., race/ethnicity), as well as stratified analyses to examine certain modifiers within strata of another. The alpha level used to define statistical significance was set to 0.05.

Finally, to determine which neighborhoods in NYC have the greatest vulnerability to heat-related mortality, we created a composite index. This index was created by calculating *z*-scores for each characteristic that was a statistically significant modifier of heat-related mortality in the regression analysis. These *z*-scores were summed (characteristics that enhanced vulnerability were added and those that were protective were subtracted) and a composite index value was assigned to each census tract. Similar approaches have been used in creating combined metrics of walkability (Marshall et al. 2009). We then assessed the degree to which living in a census tract with higher values of the index increased the risk of heat-related mortality by running a multinomial logistic regression analysis with heat wave days predicting quintiles of the composite index.

## Results

During the study period (2000–2011), there were a total of 615,088 adult deaths among residents in NYC. After restricting our data set to deaths that occurred during warm months and were of nonexternal causes, there were a total of 234,042 deaths. Less than 1% of death records (*n* = 1,470) were missing residential location and were excluded from the neighborhood characteristic analysis, for a total of 232,572 death records in the neighborhood analysis. Most deaths (73%) occurred in individuals who were > 65 years of age, and just over half of the population was female (Table 1). The daily maximum temperature ranged from 53°F to 104°F, with an interquartile range of 74–86°F. The mean daily maximum temperature over the entire period was 80°F. During the study period, 5% of the total deaths occurred during a heat wave, during 88 days (2.0%) of our time series. When the 2 days immediately following the heat wave were included, the prevalence increased to 8% of deaths, during 149 days (3.4%) of our time series. Over the study period there were a total of 31 heat waves, with a mean of 2.6 heat waves per season.

**Table 1:** Descriptive statistics for eligible adult deaths (*n* = 234,042) from nonexternal causes and study area, NYC, 2000–2011 [*n* (%) or median ± SD].

Characteristic	*n* (%) or median ± SD
Male	110,676 (47.3)
Race/ethnicity
Hispanic	37,154 (15.9)
Asian	11,485 (4.9)
White, non-Hispanic	119,469 (51.0)
Black, non-Hispanic	62,156 (26.6)
Other^*a*^	3,778 (1.6)
≥ 65 years old	171,266 (73.2)
Born outside of the USA	86,953 (37.2)
Died at home	49,221 (21.0)
Underlying cause of death
Cardiovascular disease (ICD-10, I00–I99)	95,042 (40.6)
Myocardial infarction (ICD-10, I21)	13,347 (5.7)
Congestive heart failure (ICD-10, I50)	2,226 (1.0)
COPD (ICD-10, J40–J47)	5,680 (2.4)
Built space per area of census tract (ft^2^/ft^2^)^*b*^^,^^*c*^	0.94 ± 1.27
Percent of census tract covered with trees^*b*^	16.9 ± 9.1
Percent of census tract covered with grass/shrubs^*b*^	7.1 ± 7.8
Daytime census tract surface temperature (°F)^*b*^^,^^*d*^	156.3 ± 2.7
Nighttime (0300–0500 hours) census tract air temperature (°F) ^*b*^	70.2 ± 1.0
Percent of households receiving public assistance^*b*^	5.4 ± 7.8
Percent of non-English–speaking residents^*b*^	18.3 ± 15.3
^***a***^Other, multiple race/ethnicity, or unknown. ^***b***^Based on *n *= 232,572 death records that included census tract of residence and were used in the neighborhood characteristics analysis. ^***c***^Total interior built space (ft^2^) divided by census tract area (ft^2^). This number can be greater than 1 due to the vertical dimension. ^***d***^From 18 August 2009, a period during the hottest point in the 2009 summer when cloud-free images were available.	

We considered deaths to be exposed if they occurred during the heat wave, as well as 2 days following the end of the heat wave, to examine lagged effects, in our analysis. Table 2 shows results from the individual death record characteristics examined in this analysis. Compared with deaths occurring on other warm season days, black (non-Hispanic) individuals were more likely to die during heat waves than individuals of other race/ethnicities with odds ratios (ORs) of 1.08 [95% confidence interval (CI): 1.03, 1.12] during the heat wave and 1.07 (95% CI: 1.04, 1.11) when including the 2 following days. Compared with deaths occurring on other warm season days, individuals dying during heat waves were more likely to die at home than in institutions and hospital settings: OR = 1.11 (95% CI: 1.06, 1.16) during the heat wave and OR = 1.15 (95% CI: 1.11, 1.19) when including the 2 days following the heat wave. We did not find any increased relative risk by age or sex for heat wave–related mortality, though this may be partially explained by the differential racial and sex distribution among the elderly decedents in NYC. When we stratified the elderly decedents by race/ethnicity and sex, we saw a suggestion of qualitative interaction, though neither estimate was statistically significant. When examining age ≥ 65 years as a modifier, we found an OR = 0.99 (95% CI: 0.92, 1.06) among white (non-Hispanic) individuals and an OR = 1.01 (95% CI: 0.95, 1.07) among other individuals. When stratified by sex, OR = 0.95 (95% CI: 0.90, 1.01) among men and OR = 1.03 (95% CI: 0.97, 1.10) among women, comparing those ≥ 65 years of age with those < 65 years of age. For underlying cause of death, we found that those dying of congestive heart failure were more likely to die during or immediately following a heat wave than individuals who died of other causes (OR = 1.17; 95% CI: 1.02, 1.35). However, we observed that deaths due to myocardial infarction (and all cardiovascular disease) were less likely to occur during or immediately following a heat wave than other causes of death (OR = 0.93; 95% CI: 0.87, 0.99).

**Table 2:** Relative odds of dying during or immediately following a heat wave versus dying on other days during warm months for adults who had the characteristic or cause of death, compared with adults who did not, NYC, 2000–2011.

Characteristic/underlying cause of death	All heat wave days OR (95% CI)	All heat wave days plus 2 following days OR (95% CI)
Male versus female	0.98 (0.94, 1.01)	1.00 (0.97, 1.03)
Black (non-Hispanic) versus other race/ethnicity	1.08 (1.03, 1.12)	1.07 (1.04, 1.11)
Age ≥ 65 years versus younger ages	0.99 (0.95, 1.03)	0.99 (0.96, 1.03)
Age ≥ 85 years versus younger ages	1.00 (0.96, 1.04)	1.00 (0.97, 1.04)
Born outside of the USA versus within the USA	0.99 (0.95, 1.03)	0.99 (0.96, 1.02)
Dying at home versus dying in a hospital or institution	1.11 (1.06, 1.16)	1.15 (1.11, 1.19)
Cardiovascular disease versus other underlying cause of death	0.95 (0.91, 0.98)	0.92 (0.90, 0.95)
Myocardial infarction versus other underlying cause of death	1.00 (0.92, 1.08)	0.93 (0.87, 0.99)
Congestive heart failure versus other underlying cause of death	1.18 (0.99, 1.41)	1.17 (1.02, 1.35)
COPD versus other underlying cause of death	0.99 (0.88, 1.12)	0.91 (0.83, 1.01)
COPD, chronic obstructive pulmonary disease.		

We examined which neighborhood characteristics, according to residential census tract (*n* = 2,216), might increase risk of death during a heat wave (Table 3). Some census tracts did not have sufficient data on neighborhood characteristics and were excluded from the neighborhood analysis as noted in Table 3. Individuals living in areas of the city with a relative daytime summer surface temperature above the median value had an increased relative risk of dying during a heat wave (OR = 1.05; 95% CI: 1.01, 1.09). Similarly, we found that individuals living in census tracts where the proportion of households receiving public assistance was above the median level had an increased relative risk of dying during a heat wave (OR = 1.05; 95% CI: 1.01, 1.09). We also found that individuals living in “greener” areas of the city were less likely to die during and immediately after heat waves with an OR = 0.96 (95% CI: 0.94, 0.99) for those living in census tracts with a proportion of grass and shrubs above the median value, and an OR = 0.97 (95% CI: 0.94, 1.00) for those living in census tracts with a proportion of trees above the median value. The spatial distribution of neighborhood characteristics that were statistically significant modifiers of the association between heat waves and mortality are displayed in the Supplemental Material, Figure S1.

**Table 3:** Relative odds of dying during or immediately following a heat wave versus dying on other days during warm months for adults who lived in a census tract (*n *= 2,216) with the characteristic, compared with adults who did not, NYC, 2000–2011.

Census tract characteristic^*a*^	All heat wave days OR (95% CI)	All heat wave days plus 2 following days OR (95% CI)
High amount of built space per area	0.99 (0.96, 1.03)	1.00 (0.97, 1.03)
High percent of grass/shrubs	0.98 (0.94, 1.01)	0.96 (0.94, 0.99)
High percent of trees	0.98 (0.94, 1.02)	0.97 (0.94, 1.00)
High mean temperature (based on Landsat, daytime, summer)	1.05 (1.01, 1.09)	1.04 (1.01, 1.07)
High nighttime temperature^*b*^	1.02 (0.98, 1.06)	1.02 (0.99, 1.05)
High percent of households receiving public assistance^*c*^	1.05 (1.01, 1.09)	1.04 (1.01, 1.08)
High percent of non-English speakers^*d*^	1.01 (0.97, 1.04)	1.01 (0.98, 1.04)
^***a***^All census tract characteristics were dichotomized at the median value, such that the OR compares tracts ≥ median vs. tracts < median. ^***b***^Data not available for 4 census tracts. ^***c***^Data not available for 13 census tracts. ^***d***^Data not available for 5 census tracts.		

**Figure 1:**
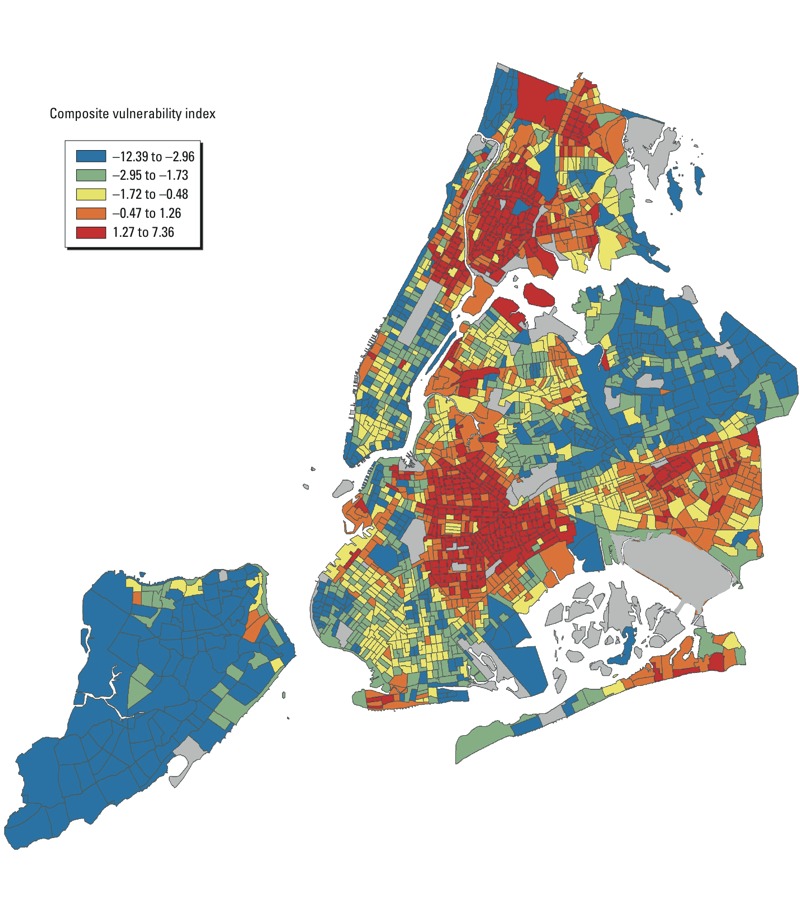
NYC census tracts according to composite heat vulnerability index. The index is composed of *z*-scores of the following variables: (+) proportion of homes receiving public assistance, (+) proportion of non-Hispanic black residents, (+) proportion of overall deaths occurring in the home, (+) relative surface temperature, (–) proportion of trees. A higher composite index score indicates a residential area with a higher risk of heat-related mortality.

**Figure 2:**
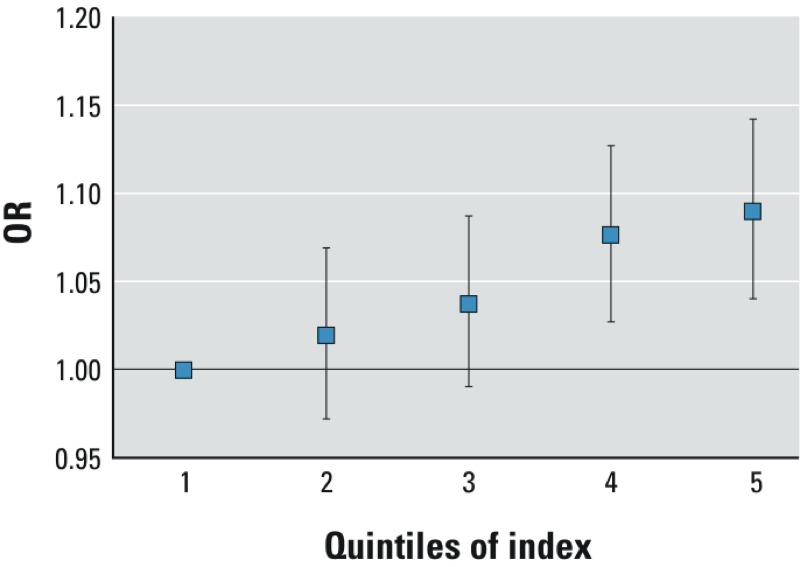
Relative odds of dying during or immediately following a heat wave by quintile of composite index. The ORs (and 95% CIs) are generated from a multinomial logistic regression model regressing the composite index on heat wave days.

We created a composite index consisting of *z*-scores of the following variables: (+) proportion of homes receiving public assistance, (+) proportion of non-Hispanic black residents, (+) proportion of overall deaths occurring in the home, (+) relative surface temperature, (–) proportion of trees. A map of census tracts according to the composite index is shown in Figure 1. When we examined the composite index as a modifier of heat-related mortality with a multinomial logistic regression, we observed a clear association (Figure 2); individuals who lived in census tracts with higher composite index scores were more likely to die during heat waves.

Our sensitivity analyses of confounding by seasonal interactions and dichotomizing modifiers at the 75th percentile showed similar results (see Supplemental Material, Tables S1–S3). Additionally, our finding of increased susceptibility among black (non-Hispanic) individuals was robust to several sensitivity analyses. When we examined race/ethnicity as a dependent variable with five categories using a multinomial logistic regression with white (non-Hispanic) as the reference, the OR for black (non-Hispanic) was 1.06 (95% CI: 1.02, 1.10). None of the other race/ethnicity categories were statistically significant modifiers (data not shown). When we further stratified our data to divide black (non-Hispanic) and Hispanic individuals into those born in the United States and those born outside the United States, the OR for black (non-Hispanic, born in the United States) was 1.08 (95% CI: 1.03, 1.14) compared with the reference category of white (non-Hispanic), and none of the other categories were statistically significant (data not shown). We next stratified our data by percentage of households receiving public assistance (using the median value as the cut point) and examined race/ethnicity again. When we examined black (non-Hispanic) race/ethnicity compared with all others, we found an increased risk in both strata: OR = 1.07 (95% CI: 1.00, 1.14) among those living in census tracts where fewer households received public assistance and OR = 1.06 (95% CI: 1.01, 1.10) among those living in census tracts where more households received public assistance.

## Discussion

We found several individual and neighborhood characteristics that were associated with an increased likelihood of death during a heat wave. Similar to other studies (Kosatsky et al. 2012; O’Neill et al. 2003), we found that the proportion of deaths at home (rather than in a hospital or institution) was increased during heat waves relative to non–heat wave warm season days; at-home deaths could be a marker of social isolation, which has been associated with an increased risk of heat-related death. During the 1995 Chicago, Illinois, heat wave, social isolation was a major predictor of death (Semenza et al. 1996). Similarly, during the 2003 Paris, France, heat wave there was an increase in deaths at home, and lack of mobility was a major risk factor (Vandentorren et al. 2006). Social isolation and lack of mobility may also explain our findings.

Consistent with findings from several studies (Greenberg et al. 1983; O’Neill et al. 2003; Schwartz 2005), we found that black (non-Hispanic) individuals were more likely to die during a heat wave than individuals of other race/ethnicity. Many studies point to the fact that in the United States, race/ethnicity is often associated with other socioeconomic disadvantages, including neighborhood contextual factors. For example, poor black individuals are more likely to live in impoverished neighborhoods than poor white individuals (Krieger et al. 1997). Indeed, we also found that living in neighborhoods that received more public assistance was associated with a higher likelihood of dying during a heat wave. Although this is only one measure of poverty, it is one that may be particularly relevant for the ability to use residential cooling, indicating that socioeconomic context is clearly an important predictor of dying during a heat wave in NYC, as has been found in other cities (Curriero et al. 2002). We attempted to disentangle the influence of race/ethnicity and poverty by examining modification by race/ethnicity separately within neighborhoods receiving more or less public assistance and found that the association with race/ethnicity was robust to neighborhood context. However, these data do not allow us to assess or control for underlying health conditions or housing factors that may vary by racial/ethnic group. For example, non-Hispanic black New Yorkers were more likely to report ever having high blood pressure and no air conditioning at home (36% and 19.5%) than New Yorkers overall (29% and 12.5%) (NYC DOHMH 2013). Thus, although socioeconomic factors may partially explain the consistent finding across studies of increased vulnerability to heat for black individuals, there may also be other behavioral, health status, and housing factors driving this.

Although findings from several studies suggest that the elderly are more susceptible to heat-related mortality (Basu 2009; Medina-Ramón et al. 2006; Stafoggia et al. 2006), we did not find evidence of a greater relative increase in mortality during heat waves among the elderly in this study, consistent with a multi-city study (O’Neill et al. 2003). Complex interactions among age, race, and other susceptibility factors may have led to confounding in our data and may partially account for our failure to find an association between age and heat-related mortality. Another hypothesis is that there may be other demographic shifts that have made the elderly in NYC more resilient in the past decade. Future analyses will examine this by looking at changes in vulnerability over time. Likewise, although heat has often been associated with cardiovascular disease (CVD) mortality (Åström et al. 2011), our results do not show evidence of increased risk of mortality for those dying of CVD. In fact, the relative odds of a death from CVD appear to be lower during heat waves in our study. Our findings do not demonstrate that persons dying of CVD do not have an increased risk of dying during heat waves, but rather that the risk of CVD-related death increases less than the risk of death from other causes.

In this study we relied on underlying cause of death information from the death certificate. Several issues may affect the validity of cause of death information and may partially explain our unexpected findings regarding CVD. First, heart disease is frequently overreported as an underlying cause of death (Coady et al. 2001) and, specifically in NYC, heart disease deaths have been overreported by as much as 91% (Agarwal et al. 2010). During 2009 and 2010 the NYC DOHMH led a hospital-level intervention that substantially improved the accuracy of cause of death reporting; however, most of our cases occurred before the intervention period (Madsen et al. 2012). It has also been reported that heart disease overreporting in NYC occurred in hospitals that tended to overrepresent white decedents (Johns et al. 2013). Systematic differences in overreporting between different racial/ethnic groups could have affected our results. Second, a limited number of studies have examined the validity of death certificate diagnosis for out-of-hospital cardiac deaths. A study of sudden cardiac disease in Minnesota indicated substantial misclassification (Iribarren et al. 1998), and another study found high sensitivity but lower specificity for coronary heart disease deaths (Folsom et al. 1987). It is possible the ascertainment of some CVD causes of death is lower for at-home deaths, but we cannot evaluate this hypothesis with the data available. Our CVD outcome includes multiple forms of the disease, including myocardial infarction and congestive heart failure, which were also evaluated separately. Finally, because our study was limited to mortality, used underlying cause of death, and did not include data on prevalent CVD among decedents, we caution against interpreting these findings as evidence that persons with CVD are not at increased risk for adverse health events during a heat wave. In fact, CVD is listed as a contributing cause of death in more than half of the NYC deaths that are directly attributable to heat illness and heat stroke during this same time period (Centers for Disease Control and Prevention 2013), and daily deaths from cardiovascular disease are directly associated with higher warm-season temperatures (Ito et al. 2011). Our findings of an increased likelihood of dying during a heat wave from congestive heart failure are in line with a study in four Italian cities demonstrating that patients hospitalized for heart failure were more susceptible to heat-related mortality than other patients (Stafoggia et al. 2008).

When considering environmental characteristics of the decedent’s neighborhood, we found that persons living in areas of the city with a higher daytime, summer surface temperature were at increased risk, and persons living in areas of the city with more green space (e.g., trees, grass, and shrubs) were at lower risk. Higher surface temperature and less vegetative cover could increase the risk of heat-related death by contributing to greater heat stress because of increased temperatures indoors and outdoors or by making travel to an air-conditioned place (e.g., NYC cooling center) more difficult during hot weather. Although the idea of increased risk in an “urban heat island” is not new, very few epidemiology studies have demonstrated this. In a previous ecologic analysis in NYC, rates of heat-related mortality among persons ≥ 65 years of age were significantly greater in neighborhoods with higher surface temperatures (Klein Rosenthal et al. 2014). In a study in Barcelona, Spain, those who lived in census tracts where residents reported a perception of little surrounding greenness were at increased risk of heat-related mortality; however, the objective measure of percent of tree cover in the census tract did not modify the association between heat and mortality (Xu et al. 2013). A recent multi-city study in the United States found that the association between temperature and mortality during warm months was stronger in areas with less green space than in other areas (Zanobetti et al. 2013). During the 2003 Paris heat wave, surface temperature around the home was a significant risk factor for death (Vandentorren et al. 2006). Our findings provide important scientific credibility for urban planning policies to increase green space. For example, NYC has a sustainability initiative to plant 1 million new trees over the next decade. We did not find a significant difference in heat-related mortality between census tracts where overnight air temperature was above the median versus below the median value. However, our measure of overnight air temperature was based on street-level monitors, which may not provide an accurate assessment of personal exposure to temperature for the majority of individuals living in the inner-urban core of NYC due to floor elevation.

The present study has a number of limitations. First, in this case-only analysis, we examined one modifier at a time. Although we attempted to disentangle such factors from each other in sensitivity analyses, further work to understand how these modifiers interact with each other is needed. In a study examining racial segregation of heat risk–related land cover (HRRLC), such as tree canopy and impervious surface area, Jesdale et al. (2013) found that non-Hispanic blacks were 52% more likely to live in HRRLC conditions than were non-Hispanic whites, and in NYC Klein Rosenthal et al. (2014) found higher surface temperatures to be correlated with increased poverty rates, impervious cover, and higher percentages of black residents in a multivariate analysis. In our data, several modifying factors were correlated or inversely correlated with each other (data not shown). For example, census tract surface temperature was inversely correlated with percent of trees in a census tract (Spearman rho = –0.51, *p* < 0.0001), but positively correlated with percent of families receiving public assistance (Spearman rho = 0.39, *p* < 0.0001) and percent of black residents (Spearman rho = 0.28, *p* < 0.0001).

As noted earlier, the lack of information on the prevalence of co-morbid conditions among decedents limits our ability to draw conclusions about co-morbid conditions as a risk factor for death during heat waves. Similarly, it would be advantageous to have individual-level socioeconomic status data, in addition to the neighborhood measure used in the present study. We used outdoor ambient temperature as a proxy for personal exposure to heat, though the amount of time that individuals spend indoors may have contributed to error in this exposure metric. Furthermore, we did not have access to data about the residential interior that could have predicted mortality, such as air conditioning use and apartment floor, which have both been cited as important predictors of heat-related mortality (Curriero et al. 2002; Vandentorren et al. 2006). A recent telephone survey indicated that approximately 11% of New Yorkers do not have a functioning air conditioner, and an additional 14% do not use their air conditioner regularly (Lane et al. 2014). Finally, the case-only method is useful for identifying groups with a greater relative risk of death associated with heat waves and does not necessarily identify groups with the greatest absolute excess risk. For example, seniors have much higher daily mortality rates and may have a higher absolute excess risk than younger adults despite not having a higher relative risk.

Even with these limitations, this is one of the few studies in the United States to comprehensively investigate vulnerability to heat-related mortality within a single, major metropolitan area. By using place-based health outcome data to inform the creation of a composite vulnerability index, we were able to visually depict neighborhoods in NYC that are at particularly high risk during heat waves. Such a tool can be used by local public health preparedness teams and a similar strategy may also be replicated in other metropolitan areas. Because there are limits to what can be done during a heat wave to reach all vulnerable populations, targeted preventive programs and policies are needed. Findings from this work are relevant for guiding such efforts to prevent heat-related deaths, including urban planning measures, public messaging during heat waves, and provision of air conditioners and electric power subsidies.

## Supplemental Material

(648 KB) PDF
