# Supplementary material for: A Case-Only Study of Vulnerability to Heat Wave–Related Mortality in New York City (2000–2011)
Source: Environ Health Perspect. 2015 Mar 17;123(7):672–8. doi: 10.1289/ehp.1408178 (PMC4492264; doi:10.1289/ehp.1408178)

Environ Health Perspect
doi: 10.1289/ehp.1408178

Note to Readers: ***EHP*** **strives to ensure that all journal content is accessible to all readers. However, some figures and Supplemental Material published in** ***EHP*** **articles may not conform to 508 standards due to the complexity of the information being presented. If you need assistance accessing journal content, please contact ehp508@niehs.nih.gov. Our staff will work with you to assess and meet your accessibility needs within 3 working days.**

Supplemental Material

A Case-Only Study of Vulnerability to Heat Wave–Related Mortality in New York City (2000–2011)

Jaime Madrigano,[1] Kazuhiko Ito,[2] Sarah Johnson,[2] Patrick L. Kinney,[3] and Thomas Matte[2]

Table of Contents

Table S1. Relative odds of dying during or immediately following a heat wave versus dying on other days during warm months for adults who had the characteristic or cause of death, compared with adults who did not, adjusted for seasonal variation[a], NYC, 2000 – 2011.

Table S2. Relative odds of dying during or immediately following a heat wave versus dying on other days during warm months for adults who lived in a census tract (n=2216) with the characteristic, compared with adults who did not, adjusted for seasonal interaction[a], NYC, 2000 – 2011.

Table S3. Relative odds of dying during or immediately following a heat wave versus dying on other days during warm months for adults who lived in a census tract with the characteristic, compared with adults who did not, using the 75th percentile cutpoint, NYC, 2000 – 2011.

Figure S1. Spatial distribution of neighborhood characteristics, NYC.

Table S1. Relative odds of dying during or immediately following a heat wave versus dying on other days during warm months for adults who had the characteristic or cause of death, compared with adults who did not, adjusted for seasonal variation[a], NYC, 2000 – 2011.

Characteristic/Underlying Cause of Death	OR (95% CI)
Male versus female	
All heat wave days	0.98 (0.94, 1.01)
All heat wave days plus 2 following days	1.00 (0.97, 1.03)
Black (non-Hispanic) versus other race/ethnicity	
All heat wave days	1.07 (1.02, 1.11)
All heat wave days plus 2 following days	1.06 (1.03, 1.10)
65 years and older versus younger ages	
All heat wave days	0.99 (0.95, 1.04)
All heat wave days plus 2 following days	0.99 (0.96, 1.03)
85 years and older versus younger ages	
All heat wave days	1.01 (0.96, 1.05)
All heat wave days plus 2 following days	1.01 (0.98, 1.05)
Born outside of the US versus within the US	
All heat wave days	0.97 (0.96, 1.04)
All heat wave days plus 2 following days	1.00 (0.97, 1.03)
Dying at home versus dying in a hospital or institution	
All heat wave days	1.10 (1.05, 1.15)
All heat wave days plus 2 following days	1.14 (1.10, 1.18)
Cardiovascular disease versus other underlying cause of death	
All heat wave days	0.95 (0.92, 0.99)
All heat wave days plus 2 following days	0.93 (0.90, 0.96)
Myocardial Infarction versus other underlying cause of death	
All heat wave days	1.02 (0.94, 1.11)
All heat wave days plus 2 following days	0.94 (0.88, 1.01)
Congestive Heart Failure versus other underlying cause of death	
All heat wave days	1.15 (0.96, 1.38)
All heat wave days plus 2 following days	1.14 (0.98, 1.32)
COPD versus other underlying cause of death	
All heat wave days	1.01 (0.89, 1.14)
All heat wave days plus 2 following days	0.92 (0.83, 1.02)

OR, odds ratio; CI, confidence interval.

[a]Models included a sine and cosine term to model season.

Table S2. Relative odds of dying during or immediately following a heat wave versus dying on other days during warm months for adults who lived in a census tract (n=2216) with the characteristic, compared with adults who did not, adjusted for seasonal interaction[a], NYC, 2000 – 2011.

Census Tract Characteristic[b]	OR (95% CI)
High built space per area	
All heat wave days	1.00 (0.96, 1.04)
All heat wave days plus 2 following days	1.01 (0.98, 1.04)
High grass/shrubs	
All heat wave days	0.97 (0.94, 1.01)
All heat wave days plus 2 following days	0.96 (0.93, 0.99)
High trees	
All heat wave days	0.98 (0.94, 1.02)
All heat wave days plus 2 following days	0.97 (0.94, 1.00)
High mean temperature (based on Landsat, daytime, summer)	
All heat wave days	1.05 (1.01, 1.09)
All heat wave days plus 2 following days	1.03 (1.00, 1.07)
High nighttime temperature[c]	
All heat wave days	1.03 (0.99, 1.07)
All heat wave days plus 2 following days	1.03 (0.99, 1.06)
High % of households receiving public assistance[d]	
All heat wave days	1.04 (1.01, 1.08)
All heat wave days plus 2 following days	1.04 (1.01, 1.08)
High % of non-English speaking[e]	
All heat wave days	1.00 (0.97, 1.04)
All heat wave days plus 2 following days	1.01 (0.98, 1.04)

OR, odds ratio; CI, confidence interval.

[a]Models included a sine and cosine term to model season. [b]All census tract characteristics were dichotomized at the median value, such that the OR compares tracts with ≥ median vs. tracts < median. [c]Data not available for 4 census tracts. [d]Data not available for 13 census tracts. [e]Data not available for 5 census tracts.

Table S3. Relative odds of dying during or immediately following a heat wave versus dying on other days during warm months for adults who lived in a census tract with the characteristic, compared with adults who did not, using the 75th percentile cutpoint, NYC, 2000 – 2011.

**Census Tract Characteristic[a]**	**OR (95% CI)**
High built space per area	
All heat wave days	0.98 (0.94, 1.03)
All heat wave days plus 2 following days	1.00 (0.97, 1.03)
High grass / shrubs	
All heat wave days	0.99 (0.94, 1.03)
All heat wave days plus 2 following days	0.97 (0.94, 1.00)
High trees	
All heat wave days	0.98 (0.93, 1.02)
All heat wave days plus 2 following days	0.98 (0.95, 1.01)
High mean temperature (based on Landsat, daytime, summer)	
All heat wave days	1.06 (1.02, 1.11)
All heat wave days plus 2 following days	1.06 (1.02, 1.09)
High nighttime temperature[b]	
All heat wave days	0.97 (0.93, 1.01)
All heat wave days plus 2 following days	0.99 (0.95, 1.02)
High % of households receiving public assistance[c]	
All heat wave days	1.04 (1.00, 1.09)
All heat wave days plus 2 following days	1.04 (1.00, 1.07)
High % of non-English speaking[d]	
All heat wave days	1.00 (0.96, 1.04)
All heat wave days plus 2 following days	1.00 (0.96, 1.03)

OR, odds ratio; CI, confidence interval.

[a]All census tract characteristics were dichotomized at the 75th percentile, such that the OR compares tracts with ≥ 75th percentile vs. tracts < 75th percentile. [b]Data not available for 4 census tracts. [c]Data not available for 13 census tracts. [d]Data not available for 5 census tracts.

Figure S1. Spatial distribution of neighborhood characteristics, NYC.

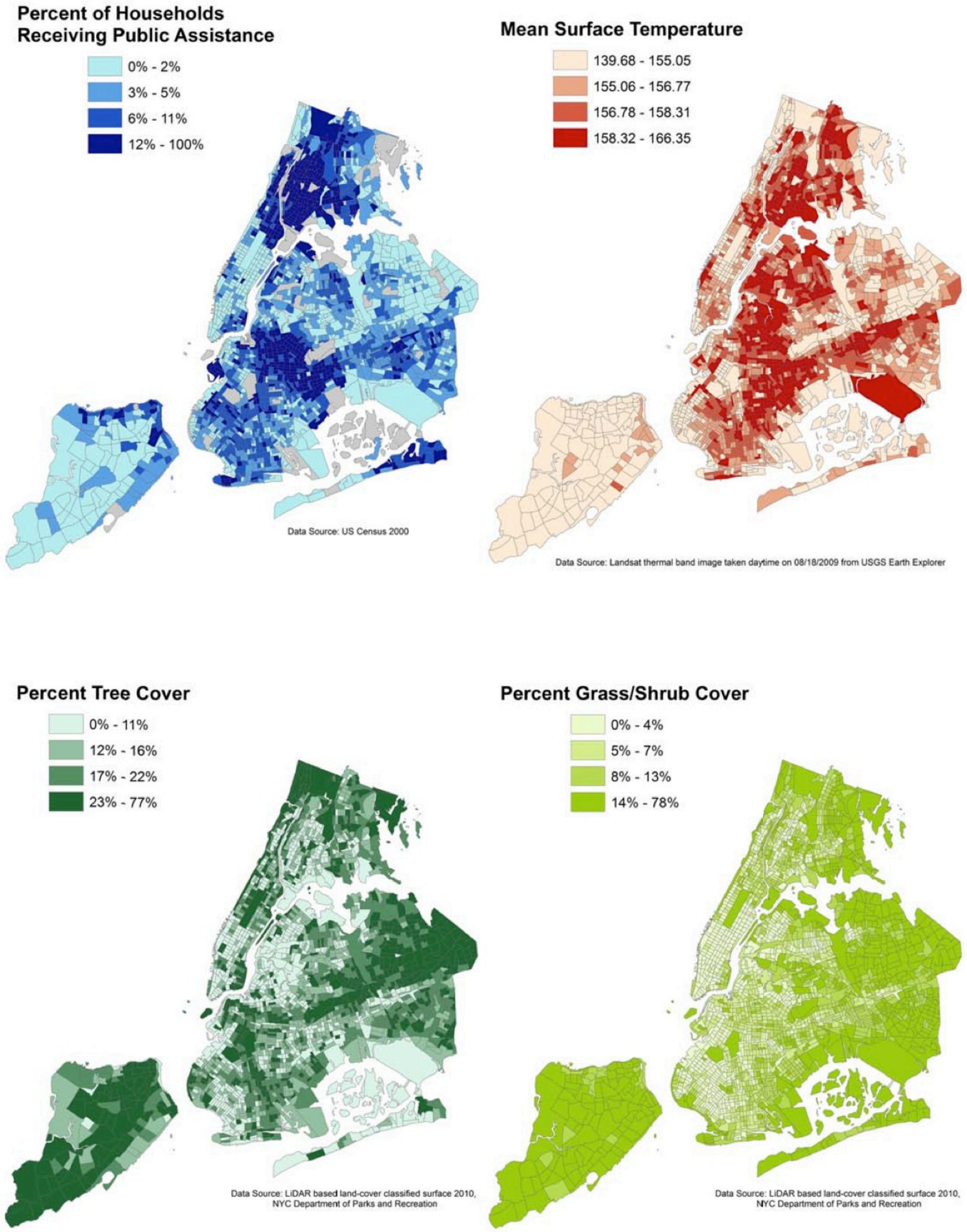

Supplement: (648 KB) PDF [file ehp.1408178.s001.acco.pdf]
